# Clinical Classification, Pregnancy Outcomes and Risk Factors Analysis of Severe Preeclampsia Complicated With HELLP Syndrome

**DOI:** 10.3389/fsurg.2022.859180

**Published:** 2022-03-14

**Authors:** Hui Huang, Bo Liu, Xia Gao, Yunju Wang

**Affiliations:** Department of Obstetrics, Renmin Hospital, Hubei University of Medicine, Shiyan, China

**Keywords:** HELLP syndrome, severe preeclampsia, clinical classification, pregnancy outcome, risk factors

## Abstract

**Purpose:**

To investigate the clinical classification, pregnancy outcomes and risk factors of pregnant women with severe preeclampsia (SPE) complicated with HELLP (hemolysis, elevated liver enzymes, and low platelets) syndrome.

**Methods:**

The clinical data of 50 pregnant women diagnosed with SPE complicated with HELLP syndrome in our hospital from January 2014 to January 2021 were retrospectively analyzed, and they were selected as the observation group. An additional 50 maternities diagnosed with preeclampsia (PE) during the same period were selected as the control group. The clinical classification and pregnancy outcomes of pregnant women in the observation group were recorded. The age and gestational age of onset of pregnancy were recorded and compared between the two groups. Univariate analysis and multivariate logistic regression model were used to analyze the risk factors for its occurrence.

**Results:**

Among the 50 maternities in the observation group, there were 10 cases of type I, accounting for 20.00%; 35 cases of type II, accounting for 70.00%; 5 cases of type III, accounting for 10.00%. Partial 33 cases, the composition ratio of 66.00%; complete 17 cases, the composition ratio of 34.00%. Among the fetuses of 50 maternities in the observation group, 35 were premature, accounting for 70.00%; 13 had fetal growth restriction, accounting for 26.00%; and 2 died during perinatal period, accounting for 4.00%. Among the 50 maternities in the observation group, 48 cases were cesarean section, the composition ratio was 96.00%; 2 cases were induced labor, the composition ratio was 4.00%; there was no natural birth, the composition ratio was 0.00%. Univariate analysis showed that age, gestational age at onset, gestational age at termination of pregnancy, HGB, LDH, ALT, AST, TBIL, PLT, PT, and FIB were all associated with the occurrence of SPE complicated with HELLP syndrome (*P* < 0.05). Multivariate logistic analysis showed that gestational age at onset, gestational age at termination of pregnancy, HGB, LDH, ALT, AST, TBIL, PLT, and FIB were independent risk factors for SPE complicated with HELLP syndrome (*P* < 0.05).

**Conclusion:**

SPE complicated with HELLP syndrome has significantly increased adverse pregnancy outcomes. Understanding its clinical classification is of great significance for the preventive application of platelet transfusion therapy and the selection of transfusion timing. Gestational age at onset and gestational age at termination of pregnancy are independent risk factors for its occurrence. Fully understanding the high-risk factors of HELLP syndrome, taking preventive measures in time, and carrying out targeted nursing can effectively improve the prognosis of pregnant women and reduce the risk of HELLP syndrome.

## Introduction

HELLP (hemolysis, elevated liver enzymes, and low platelets) syndrome is a severe form of preeclampsia, which refers to a group of clinical syndromes typically characterized by hemolysis, elevated liver enzymes and low platelets in pregnant women on the basis of gestational hypertension or severe preeclampsia (SPE) and other diseases (1). Affected patients are often accompanied by epigastric/right upper quadrant pain (40–100% incidence) (2), hypertension and proteinuria (80–85% incidence) (3), fatigue, nausea and vomiting, sudden weight gain and headache etc. HELLP syndrome occurs mostly in the second and third trimesters of pregnancy (usually between 27 and 7 weeks antenatally), and 15–30% of women present in the puerperium (usually within 7 days after delivery) (4). The pathological mechanism of the disease is not yet very clear, which may be related to placental origin (5), autoimmunity (6), mutations in coagulation factor V gene (7), fatty acid oxidation disorder symptoms (8) and so on. The incidence of HELLP syndrome is relatively low, accounting for only 0.5–0.9% of the pregnant population (the incidence in China can be about 2.5%), but the clinical manifestations are diverse, the disease develops rapidly, and poses a greater risk to maternal and child health, and the lives of mothers and children are often endangered by delayed treatment, with a high rate of maternal and child complications and death (9). Among them, the mortality rate of pregnant women is 3.4–24.2%, and the mortality rate of children in the perinatal period is as high as 7.7–60.0% (10). The vast majority of patients with HELLP syndrome should terminate their pregnancy by cesarean section after active treatment of hypertensive disorder of pregnancy (HDP). However, patients still have a 4–27% chance of recurrence when they become pregnant again. Therefore, early detection and provision of timely and effective interventions are particularly important. In this study, the clinical characteristics, pregnancy outcomes and risk factors of pregnant women with SPE complicated with HELLP syndrome were discussed and analyzed, in order to provide relevant reference materials for preventing and reducing the occurrence of HELLP syndrome.

## Materials and Methods

### Research Object

The clinical data of 50 pregnant women diagnosed with SPE complicated with HELLP syndrome in our hospital from January 2014 to January 2021 were retrospectively analyzed, and they were selected as the observation group. Inclusion criteria: patients with SPE combined with HELLP syndrome with a clear diagnosis; aged 18–50 years old; patients with complete medical records; patients or their family members who had signed the informed consent. Exclusion criteria: primary hypertension combined with pregnancy; HELLP syndrome caused by diseases other than SPE; combined with other acute and chronic life-threatening diseases not caused by SPE; combined with gestational diabetes mellitus, acute fatty liver during pregnancy, autoimmune diseases; previous hematological diseases or cardiopulmonary, hepatic and renal insufficiency; patients transferred to hospital for treatment during treatment. In addition, 50 pregnant and lying-in women diagnosed with preeclampsia (PE) during the same period were selected as the control group. An additional 50 maternal cases diagnosed with preeclampsia (PE) during the same period were selected as the control group. Inclusion criteria: those with a clear diagnosis of PE; those aged 18 to 50 years; those with complete medical records; those whose patients or their families had signed an informed consent. Exclusion criteria: primary hypertension combined with pregnancy; combined with gestational diabetes mellitus, acute fatty liver during pregnancy, autoimmune diseases; previous hematological diseases or cardiopulmonary, hepatic and renal insufficiency; patients transferred to hospital for treatment during treatment.

### Diagnostic Criteria

In accordance with the diagnostic criteria of American College of Obstetricians and Gynecologists (ACOG) for SPE (11). Hypertension: systolic blood pressure ≥160 mmHg or diastolic blood pressure ≥110 mmHg twice within 6 h of bed rest; Proteinuria: ≥5 g/24 h, or twice urinary protein (+++) at an interval of 4 h; Oliguria: 24-h urine output <500 ml; Decreased platelets (PLT): <100 × 10^9^/L; abnormal liver enzymes; persistent headache, visual disturbances, or other symptoms; Heart failure, pulmonary edema, or cyanosis; Persistent epigastric or right upper quadrant pain; Intravascular hemolysis: anemia, jaundice, elevated lactate dehydrogenase (LDH); Fetal growth restriction or oligohydramnios, etc. It met the diagnostic criteria of the University of Tennessee for HELLP syndrome (12). HELLP syndrome was considered when any one of the following was present. Hemolysis: bilirubin (BIL) ≥ 1.2 mg/dl, hemoglobin (HGB) slightly decreased, lactate dehydrogenase (LDH) > 600 U/L, broken red blood cells (RBC) on blood smear; Elevated liver enzymes: alanine aminotransferase (ALT) ≥40 U/L or aspartate aminotransferase (AST) ≥70 U/L; Decreased platelets (PLT): PLT <50 × 10^9^/L was type I, 50 × 10^9^/L≤PLT≤100 × 10^9^/L was type II, 100 × 10^9^/L<PLT<150 × 10^9^/L was type III. Partial abnormality of the above three indicators was defined as partial HELLP syndrome, and all abnormality was defined as complete HELLP syndrome.

### Research Methods

The clinical classification and pregnancy outcomes of maternities in the observation group were recorded. The maternal age, gestational age of onset and laboratory parameters were recorded and compared between the two groups. Univariate analysis was used to analyze the related factors of HELLP syndrome, and multivariate logistic regression model was used to analyze the statistically significant indexes.

### Observation Index

General information such as age, gestational age of onset, gestational age of termination of pregnancy, clinical characteristics, maternal and fetal outcomes were recorded for all mothers. Maternal RBC, HGB, LDH, ALT, AST, TBIL, blood urea nitrogen (BUN), blood calcium ion (Ca^2+^), PLT, prothrombin time (PT), prothrombin time (TT), activated partial thromboplastin time (APTT), fibrinogen (FIB) and other laboratory indicators were recorded.

### Statistical Methods

SPSS 22.0 software was used for processing, and measurement data of experimental data were expressed as mean ± standard deviation (M ± SD). One-way analysis of variance was used for comparison, and SNK-q method was used for pairwise comparison. Enumeration data are expressed as (%). Multivariate analysis was performed using a multivariate logistic regression model. The test level was α = 0.05, and *P* < 0.05 was considered statistically significant.

## Results

### Clinical Classification of 50 Maternities in Observation Group

**Figure 1:**
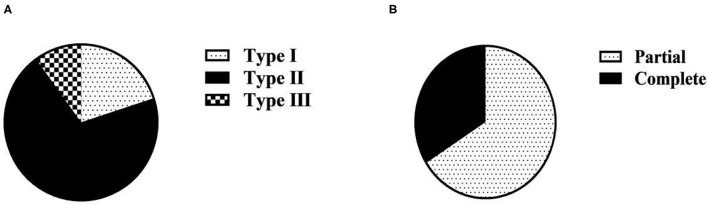
Clinical classification composition ratio of 50 maternities in the observation group (*n*, %). **(A)** Clinical classification based on PLT; **(B)** Clinical classification based on complete/partial classification.

Among the 50 maternities in the observation group, there were 10 cases of type I, accounting for 20.00%; 35 cases of type II, accounting for 70.00%; 5 cases of type III, accounting for 10.00%. Partial 33 cases, the composition ratio of 66.00%; complete 17 cases, the composition ratio of 34.00% (Table 1 and Figure 1).

**Table 1:** Clinical classification of 50 maternities in observation group (*n*, %).

	**Cases**	**Composition ratio**
**HELLP syndrome subtypes**		
Type I	10	20.00% (10/50)
Type II	35	70.00% (35/50)
Type III	5	10.00% (5/50)
**Partial/complete HELLP syndrome**		
Partial	33	66.00% (33/50)
Complete	17	34.00% (17/50)

### Pregnancy Outcomes and Mode of Delivery of 50 Maternities in Observation Group

**Figure 2:**
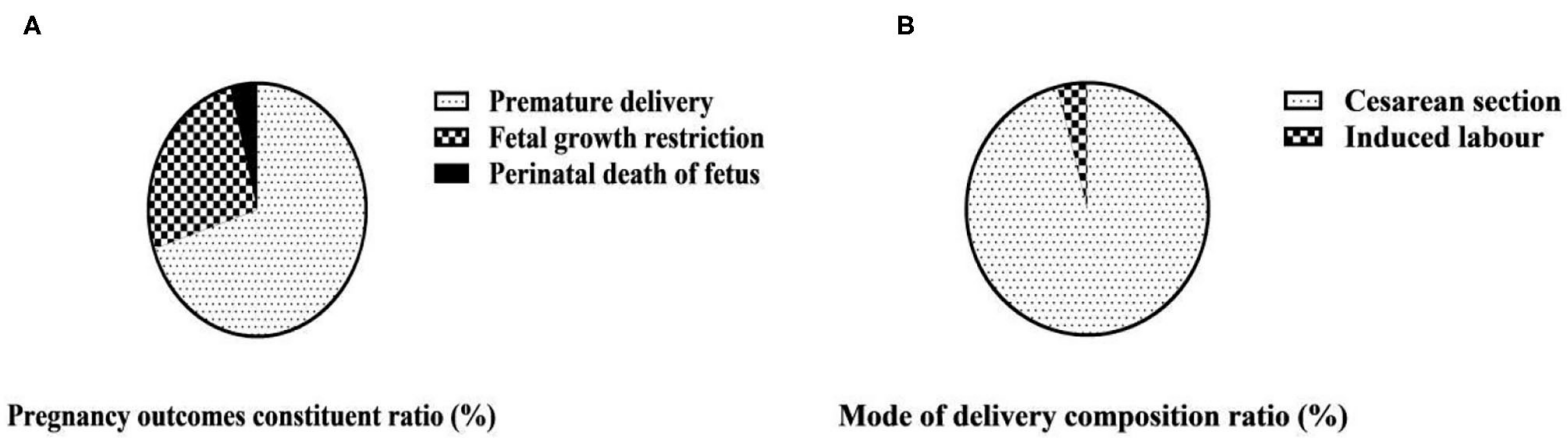
Pregnancy outcomes and mode of delivery composition ratio of 50 maternities in the observation group (*n*, %). **(A)** Pregnancy outcomes of 50 maternities in observation group; **(B)** Mode of delivery of 50 maternities in observation group.

Among the fetuses of 50 maternities in the observation group, 35 were premature, accounting for 70.00%; 13 had fetal growth restriction, accounting for 26.00%; and 2 died during perinatal period, accounting for 4.00%. Among the 50 maternities in the observation group, 48 cases were cesarean section, the composition ratio was 96.00%; 2 cases were induced labor, the composition ratio was 4.00%; there was no natural birth, the composition ratio was 0.00% (Table 2 and Figure 2).

**Table 2:** Pregnancy outcomes and mode of delivery of 50 maternities in observation group (*n*, %).

	**Cases**	**Composition ratio**
**Pregnancy outcome**		
Premature delivery	35	70.00% (35/50)
Fetal growth restriction	13	26.00% (13/50)
Perinatal death of the fetus	2	4.00% (2/50)
**Mode of delivery**		
Cesarean section	48	96.00% (48/50)
Induced labor	2	4.00% (2/50)
Natural birth	0	0.00% (0/50)

### Results of Univariate Analysis of SPE Complicated by HELLP Syndrome

Univariate analysis showed that age, gestational age at onset, gestational age at termination of pregnancy, HGB, LDH, ALT, AST, TBIL, PLT, PT, and FIB were all associated with the occurrence of HELLP syndrome (*P* < 0.05) (Table 3).

**Table 3:** Results of univariate analysis of SPE complicated by HELLP syndrome (*n*, M ± SD).

**Factors**	**Control group (*n* = 50)**	**Observation group** **(*n* = 50)**	***t-value***	***P-value***
Age (years old)	30.54 ± 5.57	34.11 ± 6.15	3.042	0.003
Gestational age at onset (week)	33.10 ± 3.97	30.54 ± 3.02	3.629	<0.001
Gestational age at termination of pregnancy (week)	34.55 ± 3.24	31.97 ± 3.93	3.582	0.001
RBC (×10^12^)	4.30 ± 2.07	3.64 ± 1.45	1.847	0.068
HGB (g/L)	114.13 ± 16.12	96.36 ± 26.30	4.073	<0.001
LDH (U/L)	482.29 ± 100.23	811.84 ± 150.21	12.904	<0.001
ALT (U/L)	37.98 ± 9.36	170.21 ± 51.30	17.930	≤ 0.001
AST (U/L)	37.78 ± 11.65	120.20 ± 59.40	9.628	≤ 0.001
TBIL (μmol/L)	18.65 ± 2.11	56.29 ± 13.16	19.969	≤ 0.001
BUN (mmol/L)	7.73 ± 3.60	8.96 ± 3.69	1.687	0.095
Blood Ca^2+^ (mmol/L)	1.98 ± 0.13	1.97 ± 0.15	0.356	0.722
PLT (×10^9^/L)	132.45 ± 38.77	72.86 ± 11.64	10.409	≤ 0.001
PT (s)	11.57 ± 3.21	20.34 ± 4.45	11.302	≤ 0.001
TT (s)	17.22 ± 1.36	17.70 ± 1.26	1.831	0.070
APTT (s)	25.33 ± 8.26	28.32 ± 7.96	1.843	0.068
FIB (mg/dl)	3.61 ± 1.65	2.98 ± 1.44	2.034	0.045

### Results of a Multifactorial Analysis of SPE Complicated by HELLP Syndrome

Multivariate Logistic analysis showed that gestational age at onset, gestational age at termination of pregnancy, HGB, LDH, ALT, AST, TBIL, PLT, and FIB were all independent risk factors for HELLP syndrome (*P* < 0.05) (Tables 4, 5).

**Table 4:** Assignment for multivariate analysis of factors.

**Factors**	**Variables**	**Assignment**
Age	X1	Continuous variable
Gestational age at onset	X2	Continuous variable
Gestational age at termination of pregnancy	X3	Continuous variable
HGB	X4	Continuous variable
LDH	X5	Continuous variable
ALT	X6	Continuous variable
AST	X7	Continuous variable
TBIL	X8	Continuous variable
PLT	X9	Continuous variable
PT	X10	Continuous variable
FIB	X11	Continuous variable

**Table 5:** Results of a multifactorial analysis of SPE complicated by HELLP syndrome.

**Factors**	***B***	***SE***	***Walds***	***P***	***OR***	***95% CI***
Age	0.220	0.231	2.449	0.117	1.246	0.792–1.960
Gestational age at onset	0.452	0.201	3.998	0.046	1.571	1.060–2.330
Gestational age at termination of pregnancy	0.310	0.124	4.577	0.032	1.363	1.069–1.739
HGB	0.534	0.211	5.997	0.013	1.706	1.128–2.579
LDH	0.320	0.124	6.698	0.008	1.377	1.080–1.756
ALT	0.298	0.117	3.989	0.047	1.347	1.071–1.694
AST	0.247	0.110	4.537	0.033	1.280	1.032–1.588
TBIL	0.312	0.102	4.604	0.032	1.366	1.119–1.668
PLT	0.453	0.118	5.254	0.021	1.573	1.248–1.982
PT	0.301	0.287	3.389	0.066	1.351	0.770–2.371
FIB	0.330	0.125	3.934	0.047	1.391	1.089–1.777

## Discussion

The incidence of HELLP syndrome is low, but it often leads to maternal placental abruption, fetal distress, and perinatal death, and has a high maternal and child mortality rate (13). The disease is typically characterized by hemolysis, elevated liver enzymes, and thrombocytopenia in pregnant women during pregnancy. The clinical manifestations are diverse, and pregnant women are often accompanied by symptoms such as fatigue and upper abdominal pain. Early detection and early treatment is the most effective and safe way to block the progression of SPE complicated with HELLP syndrome and reduce adverse outcomes. It is reported that the physiological and pathological changes of the disease are similar to those of HDP, but the initiation mechanism of its development into HELLP syndrome is still unclear. Maternal unexplained systemic small blood vessel spasm, red blood cells are squeezed and ruptured when passing through the spastic blood vessels, resulting in hemolysis (14); tissue ischemia and hypoxia lead to damage to important organs of the human body, after liver damage, liver enzymes are released, resulting in elevated liver enzymes (15); exposure of collagenous tissue after endothelial cell damage leads to platelet activation, aggregation, and excessive consumption resulting in decreased PLT (16) as its main pathological changes. Thus, the PLT count has become the most important basis for the diagnosis and staging of SPE complicated by HELLP syndrome.

The severity of the condition of maternitie complicated with HELLP syndrome is closely related to the level of serum PLT. In this study, PLT count was used as the clinical classification standard of HELLP syndrome. Among the 50 maternitie in the observation group, there were 10 cases of type I, 35 cases of type II, and 5 cases of type III. The proportion of patients with type II HELLP syndrome was 70.00%, which was much higher than the other two types. Decreased PLT is an important early warning indicator of HDP complicated with HELLP syndrome and an important manifestation of vascular endothelial injury. The main manifestation is that the lower the PLT, the more severe the vascular endothelial injury and the more severe the disease (17). In this study, the diagnosis of HELLP syndrome was used as its clinical classification criteria. Among the 50 maternities in the observation group, there were 33 cases of partial HELLP syndrome, accounting for 66.00%, and 17 cases of complete HELLP syndrome, accounting for 34.00%. This is consistent with the classification based on PLT in this study. It also suggests that in clinical, partial HELLP syndrome is more common than complete HELLP syndrome. According to the progress of diagnosis and treatment of HELLP syndrome, platelet transfusion as indicated in combination with the specific condition of the patient is an effective treatment measure. The study of the clinical classification of HELLP syndrome in this study can provide reference for the preventive platelet transfusion therapy and transfusion timing for pregnant and lying-in women with HELLP syndrome.

The occurrence of HELLP syndrome can be accompanied by irreversible damage to organs, often complicated by adverse symptoms such as placental abruption, resulting in adverse pregnancy outcomes (18). This study analyzed the pregnancy outcomes of women with SPE complicated by HELLP syndrome. Among the fetuses of 50 maternities in the observation group, 35 were premature, accounting for 70.00%; 13 had fetal growth restriction, accounting for 26.00%; and 2 died during perinatal period, accounting for 4.00%. Among the 50 maternities in the observation group, 48 cases of cesarean section, the composition ratio of 96.00%; 2 cases of induced labor, the composition ratio of 4.00%; no natural delivery. Early onset and rapid progression of HELLP syndrome can often lead to preterm delivery, and in more severe cases, it can lead to placental decline, inadequate placental blood and oxygen supply, fetal growth restriction and neonatal asphyxia, which can seriously affect fetal growth and development and increase fetal perinatal mortality (19). The mode of delivery is related to the severity of the disease and the gestational age of onset. If the condition of maternities is serious, cesarean section is often chosen to terminate the pregnancy; if the condition is mild and the intrauterine condition of the fetus is good, labor can also be induced appropriately, but the changes in the condition should be closely monitored during the process of labor induction (20).

In the risk factor analysis of this study, gestational age at onset, gestational age at termination of pregnancy, HGB, LDH, ALT, AST, TBIL, PLT, and FIB were all independent risk factors for HELLP syndrome. Analysis of the reasons, HELLP syndrome onset early in gestation, often lead to premature birth and perinatal death. The gestational age of pregnancy termination can affect the growth of the fetus in the uterus, and premature or untimely termination of pregnancy can affect HELLP syndrome. For maternities whose gestational age is >34 weeks, cesarean section is preferred, and pregnancy is terminated in time; for those whose gestational age is <34 weeks, active antispasmodic, antihypertensive and other treatments are required, or the pregnancy should be terminated within 4 days of expectant management (21). HGB is a specialized protein that transports oxygen in erythrocytes, and reduction can lead to tissue hypoxia, which is a risk factor for the development of HELLP syndrome (22). The more severely damaged the liver and the higher the serum LDH, ALT, AST and TBIL levels, the more likely HELLP syndrome will develop (23). When hemolysis occurs, LDH release is increased and serum LDH levels are elevated, affecting HELLP syndrome (24). Platelets have the effect of promoting hemostasis and can protect the endothelium of small blood vessels in the body from damage. When PLT is lowered, the protective effect of microvessels is weakened, resulting in HELLP syndrome. FIB is a glycoprotein that plays an important role in human coagulation and hemostasis, and can sensitively reflect the disorder of the coagulation system. When FIB is low, there is a risk of massive bleeding and HELLP syndrome is prone to occur (25).

Domestic studies have also pointed out that with the increase of age, maternal body function declines, and the resistance to the outside world is weakened. Therefore, advanced pregnancy is an independent risk factor for the incidence of HELLP syndrome. In the risk factor analysis of this study, age was associated with the occurrence of severe preeclampsia complicated by HELLP syndrome, but was not an independent risk factor for it. Considering that it is related to the small sample inclusion in this study, future studies with expanded samples are needed to further clarify the above risk factors.

To sum up, SPE complicated with HELLP syndrome has significantly increased adverse pregnancy outcomes. Understanding its clinical classification is of great significance for the preventive application of platelet transfusion therapy and the selection of transfusion timing. Gestational age at onset and gestational age at termination of pregnancy are independent risk factors for its occurrence. Fully understanding the high-risk factors of HELLP syndrome, taking preventive measures in time, and carrying out targeted nursing can effectively improve the prognosis of pregnant women and reduce the risk of HELLP syndrome.

## Data Availability Statement

The original contributions presented in the study are included in the article/supplementary material, further inquiries can be directed to the corresponding authors.

## Ethics Statement

The studies involving human participants were reviewed and approved by the Ethics Committee of the Renmin Hospital, Hubei University of Medicine. The patients/participants provided their written informed consent to participate in this study.

## Author Contributions

All authors listed have made a substantial, direct, and intellectual contribution to the work and approved it for publication.

## Conflict of Interest

The authors declare that the research was conducted in the absence of any commercial or financial relationships that could be construed as a potential conflict of interest.

## Publisher's Note

All claims expressed in this article are solely those of the authors and do not necessarily represent those of their affiliated organizations, or those of the publisher, the editors and the reviewers. Any product that may be evaluated in this article, or claim that may be made by its manufacturer, is not guaranteed or endorsed by the publisher.
